# Switch from eculizumab to satralizumab in aquaporin 4 immunoglobulin G–seropositive neuromyelitis optica spectrum disorder: a case series report

**DOI:** 10.3389/fimmu.2024.1526563

**Published:** 2025-01-10

**Authors:** Hesham Abboud, Adnan Subei, Buse Sengul, Robert K. Shin, Paige Goulette, Rosemarie Walch, Jeanie Coté, Robert Pace, Ahmed Z. Obeidat, Lisa Ferayorni, Shervin Gholizadeh

**Affiliations:** ^1^ Multiple Sclerosis and Neuroimmunology Program, University Hospitals Cleveland Medical Center, Case Western Reserve University, Cleveland, OH, United States; ^2^ Neurology Consultants of Dallas, Dallas, TX, United States; ^3^ Neurology Department, Memorial Healthcare, Hollywood, FL, United States; ^4^ Departments of Neurology and Ophthalmology, University of Virginia, Charlottesville, VA, United States; ^5^ Neurology Department, Memorial Healthcare, Owosso, MI, United States; ^6^ Department of Neurology, Medical College of Wisconsin, Milwaukee, WI, United States; ^7^ Genentech, Inc., South San Francisco, CA, United States

**Keywords:** NMOSD, satralizumab, eculizumab, AQP4, case series

## Abstract

**Objectives:**

This case series describes adults with aquaporin 4 immunoglobulin G–seropositive (AQP4-IgG+) neuromyelitis optica spectrum disorder (NMOSD) who switched treatment from eculizumab to satralizumab.

**Methods:**

Case information for patients with AQP4-IgG+ NMOSD who received satralizumab for ≥6 months was obtained from US healthcare providers from April 2022 to January 2024. Patient characteristics, examination findings, diagnostic test results, treatment response, and adverse events were recorded.

**Results:**

Among the 5 patients (4 women and 1 man) included, ages ranged from 32 to 81 years and 4 patients self-identified as Black/African American and 1 as White. Time since confirmed NMOSD diagnosis ranged from 1 to 14 years. The reasons for initiating satralizumab were route of administration/patient preference (n=3) and inadequate disease control with eculizumab (n=2). The duration of satralizumab treatment was 10 to 31 months. All 5 patients were relapse-free with satralizumab, and adverse events they experienced were primarily asymptomatic laboratory abnormalities.

**Discussion:**

In this retrospective case series, satralizumab was effective and well tolerated in patients with NMOSD who switched from eculizumab due to route of administration/patient preference or inadequate disease control. These outcomes align with the long-term efficacy and safety outcomes with satralizumab in the phase 3 SAkura clinical trials.

## 1 Introduction

Neuromyelitis optica spectrum disorder (NMOSD) is an autoimmune neuroinflammatory disease that primarily affects the optic nerves and spinal cord and may lead to vision loss, motor and sensory impairment, and permanent neurological disability (1). Four therapies have been approved by the US Food and Drug Administration for the treatment of adults with aquaporin 4 immunoglobulin G–seropositive (AQP4-IgG+) NMOSD. Eculizumab (2) and ravulizumab (3) are monoclonal antibodies targeting C5 complement protein and administered by intravenous (IV) infusion; eculizumab is administered weekly for the first 5 infusions and then every 2 weeks, and ravulizumab is administered every 2 weeks for 2 infusions and then every 4-8 weeks. Inebilizumab, an anti-CD19 B-cell–depleting antibody is administered by IV infusion every 2 weeks for 2 infusions and then every 6 months (4). Satralizumab, a humanized IgG2 monoclonal recycling antibody against the interleukin 6 receptor, is subcutaneously administered every 2 weeks for 3 injections and then every 4 weeks thereafter (5).

Satralizumab was developed specifically for the treatment of NMOSD and has demonstrated safety and efficacy in patients with AQP4-IgG+ NMOSD in 2 placebo-controlled, phase 3 clinical trials (SAkuraSky [NCT02028884] and SAkuraStar [NCT02073279]) (6, 7); long-term safety and efficacy were sustained in open-label extension periods (8, 9). The phase 3 satralizumab clinical trials excluded patients treated with eculizumab before enrollment, which limits the understanding of the safety and effectiveness of switching between approved therapies. Data on NMOSD treatment transitions are limited, particularly real-world data on switches between approved therapies with different mechanisms of action, which may have clinical implications. This case series aims to illustrate the real-world experience of US patients with AQP4-IgG+ NMOSD who transitioned from eculizumab to satralizumab and to reveal insights into clinical outcomes and safety profiles observed outside of controlled trial settings.

## 2 Methods

Patient information was collected from US healthcare providers between April 1, 2022, and January 31, 2024. Patients with AQP4-IgG+ NMOSD who received eculizumab followed by satralizumab for ≥6 months were included. The term *switch* refers to the transition in maintenance treatment from eculizumab to satralizumab, with no specified minimum or maximum amount of time between treatments. All patients who met the inclusion criteria were included irrespective of the clinical outcomes or patient experience. Patients provided written consent for the publication of their case information. Patient characteristics, examination findings, diagnostic test results, treatment response, and adverse events (AEs) were recorded.

## 3 Case reports

**Figure 1:**
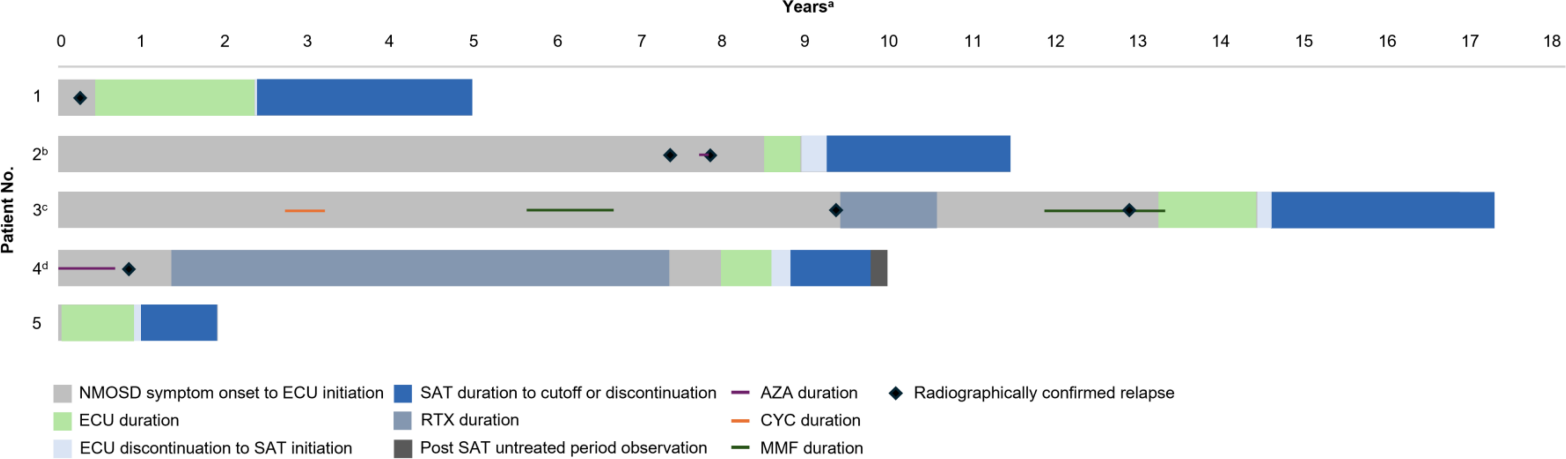
Timeline of NMOSD Treatments and Radiographically Confirmed Relapses. AZA, azathioprine; CYC, cyclophosphamide, ECU, eculizumab; MMF, mycophenolate mofetil; NMOSD, neuromyelitis optica spectrum disorder; RTX, rituximab; SAT, satralizumab. ^a^Length of bar indicates time from symptom onset to data cutoff. ^b^Patient received AZA for a brief period; the exact duration and timing are unknown. ^c^Exact date of discontinuation of ECU unknown. Patient had 4 months of interrupted SAT treatment due to insurance issues; exact timing of interruption unknown. ^d^Patient received AZA with long-term corticosteroid taper. Patient died due to stage IV cervical cancer 2 months after last dose of SAT.

Five patients (4 women and 1 man; median [range] age 56 [32-81] years) were included. Four patients self-identified as Black/African American and 1 as White (**Table 1**). The median (range) time from confirmed AQP4-IgG+ NMOSD diagnosis was 4 (1-14) years. The mean (SD) expanded disability scale score before receiving satralizumab was 4.6 (3.0). Before treatment with eculizumab, 3 patients received preventative maintenance therapies and 2 did not (**Figure 1**). The median (range) duration of eculizumab treatment was 10 (5-22) months.

**Table 1:** Demographic characteristics, clinical characteristics, and treatment history of patients with AQP4-IgG+ NMOSD who switched to satralizumab after eculizumab^a^.

Patient no.	Race	Comorbid autoimmune disease	NMOSD disease phenotype	Disease duration, years^b^	EDSS prior to satralizumab initiation^c^	Preventative therapies received before satralizumab	Duration of eculizumab treatment, months	Reason for switch to satralizumab
**1**	Black/ African American	None	TM	4	3.5	Eculizumab	22	Preferred less frequent treatment
**2**	White	None	TM	11	6.5	Azathioprine, eculizumab	5	Loss of venous access
**3**	Black/ African American	None	ON and TM	16	7.5	Cyclophosphamide, eculizumab, mycophenolate mofetil, rituximab	14	Further progression in right arm dysfunction and lack of appetite, which the patient perceived as inadequate disease control but was not a confirmed relapse
**4**	Black/ African American	Rheumatoid arthritis	ON and TM	9	5.5	Azathioprine with long-term corticosteroid taper; eculizumab, rituximab	7^d^	Clinical ON relapse (not confirmed radiographically)
**5**	Black/ African American	None^e^	ON	1	0	Eculizumab	10	Preferred self-administered therapy

AQP4 IgG, aquaporin 4 immunoglobulin G; EDSS, expanded disability scale; NMOSD, neuromyelitis optica spectrum disorder; ON, optic neuritis; TM, transverse myelitis.

a Based on data cutoff. Of the 5 patients, 4 were female and 1 was male. Ages ranged from 32 to 81 years.

b Duration calculated from time of symptom onset to data cutoff.

c EDSS or estimated EDSS.

d Exact date of eculizumab discontinuation unknown; this represents the best estimate provided by the treating healthcare provider.

e Patient has some markers of systemic lupus erythematosus (positive antinuclear antibody, positive anti–Sjögren-syndrome-related antigen A antibody, positive anti–U1-ribonucleoprotein antibody) but has not received a clinical diagnosis of systemic lupus erythematosus to date.

The primary reasons for transitioning from eculizumab to satralizumab included patient preference for administration route or frequency of satralizumab or loss of venous access (Patients 1, 2, 5) and inadequate disease control (Patients 3, 4) (**Table 1**). Patient 3 received eculizumab for 14 months, during which they experienced a progression in right arm dysfunction and a lack of appetite. The patient perceived right arm dysfunction as inadequate disease control; however, a relapse was not confirmed through radiographic evidence. Patient 4 exhibited symptoms of optic neuritis 5 months after initiating eculizumab. An MRI could not be conducted, and an ophthalmological evaluation was not obtained to confirm this as a relapse.

Median (range) time between discontinuation of eculizumab and initiation of satralizumab was 9 (2-17) weeks (**Figure 1**). The median (range) duration of satralizumab treatment was 25 (10-31) months. At the analysis cutoff date, all 5 patients had been treated with satralizumab for a duration at least as long as they had received eculizumab (Patient 5) or longer (Patients 1-4). No patients received bridging corticosteroids during the transition to satralizumab treatment, and all 5 patients received satralizumab as a monotherapy.

Patients 1, 2, 3, and 4 had ≥1 radiographically confirmed relapse before receiving eculizumab (**Figure 1**). Patient 4 experienced optic neuritis symptoms while receiving eculizumab, but this possible relapse was not confirmed with objective measures. After switching to satralizumab, all 5 patients were relapse-free and clinically stable or improved (**Table 2**).

**Table 2:** Effectiveness and safety data for satralizumab in patients with AQP4-IgG+ NMOSD who switched to satralizumab after eculizumab.

Patient no.	No. of confirmed relapses before satralizumab	No. of confirmed relapses on eculizumab	Time between eculizumab and satralizumab, weeks	Duration of satralizumab treatment, months^a^	No. of confirmed or unconfirmed relapses on satralizumab	Clinical/MRI results after satralizumab	AEs related to satralizumab^b,c^	AEs unrelated to satralizumab^b^	Concurrent disease-modifying therapies with satralizumab
**1**	1	0	2	30	0	New frontal lesion thought to be infarct and unlikely to be NMOSD lesion in brain MRI; stable cervical and thoracic spine MRI	Contact dermatitis and postinflammatory hypopigmentation of the foot; 2 instances of neutropenia that resolved (satralizumab interrupted for 1 dose on each occasion)	None	None
**2**	2	0	17	25	0	Stable cervical spine MRI; clinically improved ambulation	None	None	None
**3**	2	0	9	31^d^	0	Stable brain and cervical spine MRI	None	Anemia noted >6 months after satralizumab initiation; had been diagnosed prior to satralizumab initiation, so considered unrelated	None
**4**	1	0	13	11	0	Improvement in optic neuritis symptoms^e^; stable brain/cervical spine/thoracic spine MRI	None	Cervical cancer, death	None
**5**	0	0	5	10	0	NA	Leukopenia^f^	Elevated cholesterol, LDL, non-LDL^g^	None

AE, adverse event; AQP4 IgG, aquaporin 4 immunoglobulin G; LDL, low-density lipoprotein; NA, not available; NMOSD, neuromyelitis optica spectrum disorder.

a Satralizumab was prescribed per label instructions: a loading dosage for the first 3 administrations of 120 mg by subcutaneous injection at weeks 0, 2, and 4, followed by a maintenance dosage of 120 mg every 4 weeks (5).

b Classification of AEs considered related or unrelated to eculizumab and satralizumab was based on the opinion of the healthcare provider.

c All AEs were reported to MedWatch as required.

d Satralizumab therapy was interrupted for 4 months due to insurance coverage.

e Patient had a clinical relapse of optic neuritis while receiving eculizumab, but no MRI or ophthalmologist confirmation was available. The acute treatment and affected eye were not reported.

f Leukopenia was mild; the lowest white blood cell count was 3300/µL. Neutrophil count was never less than 1500/µL.

g The elevated cholesterol levels were not considered related to satralizumab because levels were elevated before satralizumab initiation and triglycerides were normal.

AEs related to satralizumab, as determined by their healthcare providers, were reported by 2 patients (**Table 2**). Patient 1 had neutropenia twice, at 2 months and at 1 year after initiation; on each occasion, satralizumab was temporarily interrupted for 1 dose, the neutrophil count increased at repeat testing 1 month later, and satralizumab was resumed. This patient also had contact dermatitis with postinflammatory hypopigmentation. Patient 5 had leukopenia at 1 month and 3 months after satralizumab initiation without interruption in satralizumab dosing. Patient 4 received satralizumab for 11 months with no AEs; however, the patient died 2 months after discontinuation of satralizumab due to stage IV cervical cancer.

## 4 Discussion

This case series presents the clinical courses of 5 US patients with AQP4-IgG+ NMOSD who switched from eculizumab to satralizumab in real-world practice because of preferred route/frequency of administration (including loss of venous access) or inadequate disease control with eculizumab. After switching to satralizumab monotherapy, all patients were relapse-free, and AEs were primarily asymptomatic laboratory abnormalities. At data cutoff, 4 patients were receiving satralizumab, 3 for ≥25 months. These results are consistent with a case report of a patient who had no reported relapses or AEs after switching from eculizumab to satralizumab (10).

Patients with NMOSD may switch from one treatment to another due to lack of effective disease control or AEs as well as route of administration preference, medical insurance coverage, and real-world access (11). Uncontrolled disease activity is important in predicting treatment change in patients with NMOSD (12). Switching between NMOSD treatments due to disease activity, tolerability, or nonmedical reasons is common, with reason for switch influencing risk of disease advancement (11). Understanding reasons for and outcomes after switching between approved therapies may aid in developing optimum treatment strategies in the clinical management of NMOSD.

Among a panel of NMOSD experts, the most common considerations for choosing therapies after efficacy and safety were disease or relapse severity and patient preference for treatment administration (13). Additionally, these panel experts agreed that the choice between the NMOSD therapies may be informed by patient preferences for dosing frequency and route of administration, along with acceptance of safety risks (13). In this case series, patients 1 and 5 switched to satralizumab due to a preference for the less-frequent treatment and self-administered therapy, respectively. In a US cross-sectional survey of people with NMOSD, the highest proportions of patients were concerned with treatment effectiveness and AEs; administration concerns were also reported, including discomfort during administration and treatment inconvenience (14).

High rates of disease reactivation and relapse have been documented in the first 3 months following discontinuation of eculizumab (15). In our case series, the interval between eculizumab discontinuation and satralizumab initiation ranged from 2 to 17 weeks, yet no relapses were observed after discontinuation of eculizumab. Literature suggests that when switching between approved therapies, the new treatment can be started immediately after cessation of the previous one, taking into account the mechanism and onset of efficacy of the new treatment (13). In this series, satralizumab was effective, regardless of the duration between eculizumab discontinuation and satralizumab initiation and whether patients were clinically stable or had experienced a possible relapse during eculizumab treatment. It is important to note that patient 3 switched due to their subjective sense of disease progression but without objective evidence of relapse. In that case, measuring serum CH50 may have been helpful to evaluate the biological efficacy of eculizumab but it was not done, limiting a definitive conclusion regarding disease activity. Future eculizumab switches due to efficacy concerns could benefit from such measurement. Importantly, switching from eculizumab to satralizumab did not increase infection risk or cause unexpected AEs.

This study’s limitations include the small number of patients, partially missing data, lack of a control group, and retrospective design. The aim of this report was to enhance real-world data on treatment options in NMOSD; our findings should not be construed or interpreted as evidence of superiority or inferiority of either treatment in the absence of head-to-head clinical trials. We understand the importance of larger data sets in strengthening the conclusions drawn. Currently, there are no publications regarding switches from eculizumab to satralizumab or from eculizumab to any other treatment in NMOSD. Given the rarity of these switches—owing largely to the success of eculizumab in managing the disease—we believed it was imperative to report these cases. This information could potentially assist treating physicians in making informed decisions. We intend to report additional cases as they occur, though such switches remain uncommon. Future studies should also evaluate biomarker changes during treatment switches. Nevertheless, this case series provides valuable real-world data on patients with AQP4-IgG+ NMOSD who switched from eculizumab to satralizumab.

## 5 Conclusions

In this retrospective case series, satralizumab was effective and well tolerated in patients with NMOSD who switched from eculizumab treatment. All patients were relapse-free, and no major AEs related to satralizumab were reported. These outcomes align with the long-term efficacy and safety outcomes observed in satralizumab clinical trials.

## Data Availability

The datasets presented in this article are not readily available because of ethical and privacy restrictions. Requests to access the datasets should be directed to the corresponding author.
